# Supplementary material for: Multiple Sclerosis Onset before and after COVID-19 Vaccination: Can HLA Haplotype Be Determinant?
Source: Int J Mol Sci. 2024 Apr 22;25(8):4556. doi: 10.3390/ijms25084556 (PMC11050425; doi:10.3390/ijms25084556)
Supplement: Supplementary file 1 [file ijms-25-04556-s001.zip › ijms-2944316-supplementary.pdf]

Supplementary Table S1: Univariate analysis post PSM regarding the effect of post-vaccine onset* of MS with the EDSS at presentation, at relapse and recovery after relapse. *Considering a maximum of 1 month between vaccination and MS onset

	**Post-vaccine diagnosis**	**All patients diagnosed b/w 2018 and 2022**	**p-value***
Number of patients	11	11	
Onset EDSS - median (IQR)	2.0 (1.13 – 2.38)	2.0 (1.13 – 2.38)	0.514
EDSS After Relapse - median (IQR)	1.0 (0.25 – 1.50)	1.0 (0.25 – 1.50)	0.812
Recovery			0.735
None (%)	1 (9.1%)	1 (9.1%)	
Partial (%)	7 (63.6%)	6 (54.5%)	
Full (%)	3 (27.2%)	4 (36.4%)	

Supplementary Table S2: Comparison between patients with post-vaccine and non-vaccine related new diagnosis of MS considering a maximum of 2 months between Sars-CoV-2 vaccination and demyelinating event onset.

	**Post-vaccine new diagnosis of MS**	**Non-vaccine related new diagnosis of MS**	**p-value***
Number of patients with new diagnosis of MS	18	248	
Women (%)	14/18 (77.8%)	174/248 (70.2%)	0.574
Age in years - median (IQR)	33.0 (26.00 – 40.00)	33.0 (26.00 – 40.00)	0.156
Family History of MS (%)	1/18 (5.6%)	17/248 (6.9%)	1.000
Autoimmune comorbidity (%)	0/18 (0.0%)	25/248 (10.1%)	0.331
Time from Onset to Diagnosis - median (IQR)	4.0 (2.00 – 7.00)	4.0 (2.00 – 8.00)	0.907
Type of onset			0.882
Optic neuritis (%)	2/18 (11.1%)	48/248 (19.4%)	
Brain stem (%)	5/18 (27.8%)	48/248 (19.4%)	
Cerebellum (%)	0/18 (0.0%)	4/248 (1.6%)	
Spinal Cord (%)	6/18 (33.3%)	66/248 (26.6%)	
Supratentorial (%)	1/18 (5.6%)	29/248 (11.7%)	
Multisystemic (%)	4/18 (22.2%)	53/248 (21.4%)	
Oligoclonal bands (%)	13/16 (81.3%)	209/233 (89.6%)	0.538
EDSS at onset - median (IQR)	2.0 (1.50 – 2.50)	2.0 (1.50 – 2.50)	0.511
EDSS After Relapse - median (IQR)	1.0 (1.00 – 1.25)	1.0 (0.00 – 1.50)	0.418
Clinical recovery after pulsed steroid therapy			0.201
None (%)	2/18 (11.1%)	15/248 (6.0%)	
Partial (%)	12/18 (66.7%)	151/248 (60.9%)	
Full (%)	4/18 (22.2%)	82/248 (33.1%)	
MRI Brain stem (%)	8/18 (44.4%)	101/248 (40.7%)	0.914
MRI Cerebellum (%)	5/18 (27.8%)	71/248 (28.6%)	1.000
MRI Spinal cord (%)	13/18 (72.2%)	179/248 (72.2%)	0.954
MRI Infratentorial (%)	11/18 (61.1%)	130/248 (52.4%)	0.595
MRI number of supratentorial lesions - median (IQR)	5.0 (3.00 – 9.00)	5.0 (3.00 – 9.00)	0.396
MRI Contrast Enhancement (%)	13/18 (72.2%)	131/248 (52.8%)	0.168
Highly active efficacy therapy (%)	10/13 (76.9%)	112/243 (46.1%)	0.055
Smoke (%)	4/18 (22.2%)	83/248 (33.5%)	0.444

Vitamin D - median (IQR)	23.9 (17.55 – 30.76)	23.9 23.9 (17.55 – 30.76)	0.123
BMI (kg/m2) - median (IQR)	23.6 (21.30 – 26.23)	23.6 (21.30 – 26.23)	0.995
EBV positive serology (%)	17/18 (94.4%)	201/248 (81.0%)	0.283

Supplementary Table S3: Descriptive HLA-DRB1 Haplotypes of 17 patients with disease onset in the pre-COVID era (control group 1), 18 patients with disease onset in the post-COVID but not-vaccine-related (control group 2), and all 14 patients with a post-vaccine new diagnosis of MS (cases).

**HLA-DRB1 Haplotypes**		
before-COVID19 era	COVID19-era	
	Post-vaccine	Not vaccinated
DRB1*13/DRB1*13	DRB1*13/DRB1*13	DRB1*03/DRB1*16
DRB1*03/DRB1*07	DRB1*01/DRB1*01	DRB1*10/DRB1*11
DRB1*01/DRB1*07	DRB1*03/DRB1*14	DRB1*13/DRB1*13
DRB1*11/DRB1*14	DRB1*04/DRB1*13	DRB1*01/DRB1*04
DRB1*01/DRB1*16	DRB1*01/DRB1*04	DRB1*08/DRB1*11
DRB1*03/DRB1*04	DRB1*03/DRB1*07	DRB1*03/DRB1*04
DRB1*15/DRB1*16	DRB1*03/DRB1*07	DRB1*01/DRB1*16
DRB1*07/DRB1*13	DRB1*08/DRB1*13	DRB1*03/DRB1*11
DRB1*15/DRB1*14	DRB1*15/DRB1*15	DRB1*11/DRB1*16
DRB1*03/DRB1*07	DRB1*03/DRB1*11	DRB1*08/DRB1*13
DRB1*03/DRB1*11	DRB1*15/DRB1*15	DRB1*08/DRB1*13
DRB1*13/DRB1*04	DRB1*07/DRB1*15	DRB1*10/DRB1*13
DRB1*04/DRB1*11	DRB1*03/DRB1*11	DRB1*11/DRB1*12
DRB1*11/DRB1*15	DRB1*03/DRB1*07	DRB1*07/DRB1*07
DRB1*03/DRB1*16	DRB1*01/DRB1*01	
DRB1*11/DRB1*11	DRB1*15/DRB1*15	
DRB1*07/DRB1*13	DRB1*01/DRB1*01	
	DRB1*11/DRB1*14	
